# Transcriptional dissection of pancreatic tumors engrafted in mice

**DOI:** 10.1186/gm544

**Published:** 2014-04-16

**Authors:** Raquel Martinez-Garcia, David Juan, Antonio Rausell, Manuel Muñoz, Natalia Baños, Camino Menéndez, Pedro P Lopez-Casas, Daniel Rico, Alfonso Valencia, Manuel Hidalgo

**Affiliations:** 1 Gastrointestinal Cancer Clinical Research Unit, Clinical Research Programme, Spanish National Cancer Research Center (CNIO), 28029 Madrid, Spain; 2 Structural Biology and Biocomputing Programme, Spanish National Cancer Research Center (CNIO), 28029 Madrid, Spain; 3 SIB Swiss Institute of Bioinformatics, Vital-IT Group, 1015 Lausanne, Switzerland; 4 Institute of Microbiology, Lausanne University Hospital and University of Lausanne, 1011 Lausanne, Switzerland

## Abstract

**Background:**

Engraftment of primary pancreas ductal adenocarcinomas (PDAC) in mice to generate patient-derived xenograft (PDX) models is a promising platform for biological and therapeutic studies in this disease. However, these models are still incompletely characterized. Here, we measured the impact of the murine tumor environment on the gene expression of the engrafted human tumoral cells.

**Methods:**

We have analyzed gene expression profiles from 35 new PDX models and compared them with previously published microarray data of 18 PDX models, 53 primary tumors and 41 cell lines from PDAC. The results obtained in the PDAC system were further compared with public available microarray data from 42 PDX models, 108 primary tumors and 32 cell lines from hepatocellular carcinoma (HCC). We developed a robust analysis protocol to explore the gene expression space. In addition, we completed the analysis with a functional characterization of PDX models, including if changes were caused by murine environment or by serial passing.

**Results:**

Our results showed that PDX models derived from PDAC, or HCC, were clearly different to the cell lines derived from the same cancer tissues. Indeed, PDAC- and HCC-derived cell lines are indistinguishable from each other based on their gene expression profiles. In contrast, the transcriptomes of PDAC and HCC PDX models can be separated into two different groups that share some partial similarity with their corresponding original primary tumors. Our results point to the lack of human stromal involvement in PDXs as a major factor contributing to their differences from the original primary tumors. The main functional differences between pancreatic PDX models and human PDAC are the lower expression of genes involved in pathways related to extracellular matrix and hemostasis and the up- regulation of cell cycle genes. Importantly, most of these differences are detected in the first passages after the tumor engraftment.

**Conclusions:**

Our results suggest that PDX models of PDAC and HCC retain, to some extent, a gene expression memory of the original primary tumors, while this pattern is not detected in conventional cancer cell lines. Expression changes in PDXs are mainly related to pathways reflecting the lack of human infiltrating cells and the adaptation to a new environment. We also provide evidence of the stability of gene expression patterns over subsequent passages, indicating early phases of the adaptation process.

## Background

Patient-derived xenograft (PDX) models are becoming a common platform for research and clinical purposes [1]. The establishment of PDX models to study cancer biology and pharmacology is a common practice that has been successfully applied to many cancer types [2-5]. Xenografting of human primary carcinomas is in fact the only method currently available that permits the propagation of a significant proportion of carcinomas [6-8] and has many advantages over tumor-derived cell lines maintained *in vitro*[9-11]. Both cell lines and PDX models permit the removal of contaminating non-neoplastic human cells from the human tumors. However, the tissue architecture is only partially maintained in PDXs [2,11,12] with mouse stromal cells substituting for human stromal cells [13]. In general, the results obtained using PDX models in mice show better preclinical and clinical concordance than those from cell lines [11,14].

Pancreas ductal adenocarcinoma (PDAC) is usually diagnosed in advanced stages after it has metastasized to regional lymph nodes, liver or lung [15] and the median survival after diagnosis is approximately 8 months [16]. PDAC is notorious for how difficult it is to obtain biological material to study the disease. In addition, standard treatments have a very low percentage of success and the short survival time of the patients makes it challenging to search for alternative therapies [17]. For these reasons, PDX models are particularly attractive for studying PDAC.

Despite their advantages over cell lines, fresh tumors xenografted in mice show differences from the original primary tumors [11]. For example, the proportion of murine stromal cells in PDAC PDXs is lower than the proportion of human stromal cells in the original primary tumors (our experimental observations). Thus, caution should be taken when interpreting the results obtained using these models. A study by Gadaleta *et al.*[18] analyzed the so-called 'pancreas expression space' by combining publicly available gene expression array datasets studied with the Affymetrix Human Genome U133 Plus 2.0 Array platform. This pancreas expression space included healthy pancreatic tissue, human primary pancreas cancer, non-tumoral tissue adjacent to tumor, tumor-derived cell lines and PDX models. These authors applied a statistical method (principal component analysis) to explore how the different samples clustered in the two first principal components [18]. Their main findings were that (1) non-tumoral tissue adjacent to tumor was different to healthy pancreatic tissue, (2) primary tumors and tumor adjacent samples clustered together, and (3) PDXs and cell lines clustered in two other groups. One of their main conclusions was 'that ectopic subcutaneous xenografts and cell line models do not effectively represent changes occurring in pancreatic cancer'. This work highlighted the importance of understanding better the extent to which the mouse environment is altering the gene expression of the implanted human tumoral cells.

Our central goal was to understand how the ectopic xenograft mouse environment affects the expression phenotype of PDAC cells. We developed a robust analysis protocol to explore the PDAC expression space, including human primary tumors, tumor-derived cell lines and PDX models. We used expression data from primary tumors, PDX models and cell lines derived from PDACs. In addition, we used hepatocellular carcinoma (HCC) to study if PDXs and cell lines share any characteristics with the original primary tumors. The study shows that the gene expression profiles of PDAC and HCC PDX models conserved substantial similarity with their original primary tissues, while all cell lines clustered together, independently of their tissues of origin. However, we also identified several biological pathways that showed differential expression between PDX models and tumors, such as extracellular matrix organization, likely due to replacement of the human stroma by murine elements, and up-regulation of cell cycle and DNA replication that are indicative of higher proliferation. We also show that these changes happened at initial stages after engrafting and were stable over different passages.

## Materials and methods

### Generation of PDX models and sample processing

We performed gene expression profiling for 35 new PDX models of PDACs xenografted in nude mice and 2 human primary tumor samples. PDX models were established following an already described protocol [1]. Mice used in this research have been treated humanely according to the regulations laid down by the CNIO Bioethics Committee and the relevant EC guidelines (directive 86/609/EEC), with due consideration to the alleviation of distress and discomfort. RNA was extracted using Qiagen RNeasy Mini kits (QIAGEN Inc., Valencia, CA, USA) and was hybridized in GeneChip® Human Genome U133 Plus 2.0 Array, Affymetrix (Santa Clara, CA, USA). The data generated in this publication have been deposited in NCBI’s Gene Expression Omnibus (GEO) and are accessible through GEO Series accession number GSE51798.

### Public datasets

In addition to the new gene expression profiles specifically obtained for this study, we retrieved data from GEO series [19,20] that were hybridized in the same platform as our models (Human Genome U133 Plus 2.0 Array). We also added the data from a dataset with 18 expression profiles from PDX models (GSE9599) previously described [21]. We downloaded expression data for 51 original tumor samples available from Badea *et al.*[22] and Pei *et al.*[23] published in datasets GSE15471 and GSE16515. We also used gene expression profiles of 22 cell lines from Maupin *et al.*[24] and 19 from Collisson *et al.*[25]. These two datasets have data from 11 cell lines in common. All samples corresponded or were derived from adenocarcinomas.

In addition, we compiled an HCC dataset with 182 expression profiles, including data from 42 HCC PDX models [26], 32 cell lines [26,27] (these two datasets have data from 5 cell lines in common) and 108 primary HCC [26,28-31] samples. All samples corresponded to carcinomas, except two cell lines (HuH-6 and NCI-H684) that were derived from hepatoblastomas. All datasets used to create gene expression spaces are summarized in Table 1.

**Table 1:** Microarray datasets used in this study

	**Pancreas**		**Liver**	
	**Samples**	**GEO accession**	**Samples**	**GEO accession**
PDX models	53	GSE9599 [21], **GSE51798**	42	GSE6465 [26]
Cell lines	41 (11 repeated)	GSE17891 [25], GSE21654 [24]	32 (5 repeated)	GSE6465 [26], GSE36133 [27]
Primary Tumors	53	GSE15471 [22], GSE16515 [23], **GSE51798**	108	GSE6465 [26], GSE6222 [28], GSE9843 [29-31]

Number of samples and GEO accession for all datasets used in the study to create gene expression spaces. The new dataset that was generated in this study is indicated in bold.

We also downloaded two datasets of expression profiles of pancreatic primary tumors and metastases, GSE42952 [32] and GSE34153 [33]. GSE42952 was normalized and processed with the same protocol as the rest of the samples in the HGU133 Plus 2.0 Array platform. The GSE34153 dataset corresponds to the Agilent 4x44 K whole human genome array platform and we used the normalized data available in GEO series.

### Analysis protocol

We used the frozen robust multiarray analysis (fRMA) method for preprocessing and normalization of microarrays. fRMA pre-processes arrays individually and allows addition of new samples to a previously normalized dataset [34], a feature that is very convenient when new samples are included in a pre-existing dataset. We then utilized the Gene Expression Barcode method included in the fRMA R package that converts normalized expression intensities into expression binary calls (silenced or expressed). The main benefit of this approach is that it minimizes batch effects and reduces noise [35]. As we were not using expression intensities but yes/no expression calls, we used the multiple correspondence analysis (MCA) method [36] to explore the pancreas expression space. MCA is equivalent to principal component analysis (PCA) when working with qualitative data instead of continuous variables. In this work we have adapted a methodological protocol, previously applied to multiple sequence alignments, for the automatic extraction of relevant signatures from MCA results [37]. In brief, our protocol performs a MCA on a vectorial representation of multiple 'barcoded' microarray data in a high dimensional space. MCA produces a new multidimensional space so that the accumulated variance of the coordinates of every probe in a subset of dimensions is optimal. The MCA space is reduced to a low dimensional one preserving most of the original information but filtering the main sources of noise. Our protocol establishes a low dimensional space composed of those dimensions that incorporate results in a statistically significant increment of the information. The number of informative dimensions (those that explain most of the total variance) is selected according to the *P*-value of a Wilcoxon test between contiguous axes. Additional axes are included in the expression space if the *P*-value is lower than 0.01. Robust unsupervised k-means clustering is performed iteratively on this reduced space (defined by the informative dimensions) for a range of pre-specified numbers of groups (from 2 to 50). Finally, optimal clustering solutions are detected as those maximizing the Calinsky’s and Harabsz’s (CH) index [37].

### Estimation of non-tumoral component and correction for its effect

We measured the proportion of stromal and infiltrating immune cells using ESTIMATE [38], a gene expression signature-based method that estimates tumor purity from gene expression data. ESTIMATE scores were calculated using the original R code from the authors using the default parameters for the Affymetrix arrays. To remove the contribution of human non-tumoral infiltrating (hNTI) cells to the PDX primary tumor expression space, we first generated an expression space without the signature genes used by ESTIMATE. Then, we removed the gene expression signal directly relating to the effect of hNTI cells from remaining genes using simple linear regression models. We fitted two linear models, one for the first component and the other for the second component, using in both cases the ESTIMATE score as the explanatory variable (R^2^ = 0.04, *P*-value = 0.002 and R^2^ = 0.83, *P*-value <2.2 × 10^-16^, respectively; Additional file 1). Next, we removed the non-tumoral trend of each component using the corresponding linear regression models. The regression residuals of each model were used to generate a new gene expression space corrected for the contribution of non-tumoral infiltrating cells.

### Pathway enrichment analyses

To study differences between original patients’ tumors and PDX models we calculated the number of times that each gene was called as 'expressed' in each group, or its 'expression frequency'. Here we considered that a gene was expressed if any of the probes detecting that gene in the microarray was called as 'expressed' according to the Barcode method. The difference in expression frequency for each gene between each group was obtained by subtracting the gene expression frequency in PDX from the gene expression frequency in primary tumors. We ranked the values of differences in expression frequency and performed gene set enrichment analyses (GSEAs) with the GseaPreranked tool of the GSEA software [39] using as gene sets the pathways annotated in Reactome [40]. A total of 621 pathways were testable after the default filtering step (at least 15 genes and no more than 500 genes in each gene set). We grouped together similar significant pathways (false discovery rate (FDR) <0.05) as ‘functional groups’, guided by the Reactome hierarchy. For comparisons of primary tumors and metastases [32,33], we used the normalized expression matrices as input for GSEA to test if functional groups were differentially expressed between metastases and primary tumors. We used *t*-test as the metric for ranking genes.

### Differential expression between different passages

We used the Limma package [41] to test differential gene expression between four patients’ tumors and PDX models developed from them at different passages [42]. We created a linear model with information about number of passages: primary original tumor (F0), 5th passage (F5) and 10th passage (F0), using each patient as blocking information. Then we contrasted F0 versus F5, F5 versus F10 and F0 versus F10. After each contrast analysis, we ranked genes according to their t-statistic and used the GseaPreranked tool (see above) to analyze if the expression levels of the genes changed throughout passages.

## Results and discussion

### Pancreas and liver cancer gene expression spaces

**Figure 1:**
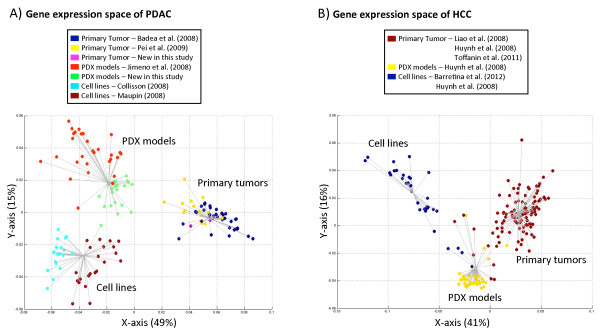
**MCA gene expression spaces from PDAC and HCC. (A)** PDAC gene expression space. **(B)** HCC gene expression space. The percentage of variance explained by each axis is indicated between parentheses. Each point represents the expression pattern from a sample. Grey lines represent the resulting clusters from k-means clustering for k = 3 (selected as the optimal clustering result; see Materials and methods). Each line joins a sample with the centroid of its cluster. **(A)** In pancreas, all PDX models and cell lines were derived from adenocarcinomas. **(B)** In liver, all samples came from carcinomas, except two cell lines that were derived from hepatoblastomas. See text for details. See Figures S1 and S2 in Additional file 1 for additional results using PCA and hierarchical clustering.

We compiled a PDAC gene expression dataset comprising a total of 147 PDAC and PDAC-derived profiles, including 53 PDX models, 53 whole-tissue primary PDACs and 41 cell lines (Table 1), in part collected from databases and in part derived for this study. We processed all data using the same analysis protocol, transforming the expression intensities into expression calls [34,35]. Then, we used our MCA implementation to explore quantitatively the transcriptional space of biological samples based on their gene expression binary profiles (expressed or not expressed; see Materials and methods for details). Figure 1A shows the first two dimensions of the PDAC expression space explaining 64% of the variance (49% by the first axis and 15% by the second). The distribution of the samples in the first and second axes is significantly different (*P*-value = 1.7 × 10^-4^, Wilcoxon test), while adding a third dimension to the expression space does not result in a significant gain of information (*P*-value = 0.08; significance threshold = 0.01).

The MCA results clearly show that the human tumor samples, PDX models and cell lines were three distinct groups (Figure 1A) based on the transcriptional profile of the complete genome as represented in the arrays. In fact, k-means clustering of the space created by the MCA found an optimal solution that corresponds to three clusters (k-means = 3, CH index = 199.541): (1) primary original tumors, (2) PDXs and (3) cell lines. Similar results were obtained when analyzing the normalized intensities with PCA or by hierarchical clustering of the expression calls (Figures S1A and S2A in Additional file 1). Therefore, based on expression profiles, PDX models and cell lines, considered globally, are as different from the original tumors as they are from each other.

To compare our results for PDAC with other cancer types, we used publicly available HCC microarray data, for which we could gather a sufficiently large collection of human primary tumors, PDX models and cell lines. We were able to compile a dataset with a total of 182 expression profiles from HCC, including 42 HCC PDX models, 32 cell lines and 108 primary HCCs. We applied the same analysis protocol used for the PDAC data. Figure 1B shows the HCC transcriptional space generated by the first two dimensions of the MCA (Wilcoxon test *P*-value = 1.6 × 10^-7^), which explain 57% of the variance (41% by the first axis and 16% by the second). HCC PDX models and cell lines were also located in different areas of the HCC expression space (Figure 1B). Indeed, the k-means clustering of the space also had an optimal solution corresponding to three clusters (k-means = 3, CH index = 236.304): (1) human primary tumors, (2) PDX models and (3) cell lines. Similar clustering of the samples was obtained using alternative methods (PCA of expression intensities and hierarchical clustering of expression calls; Figures S1B and S2B in Additional file 1). The results obtained with the HCC samples were in agreement with the previously shown PDAC clusters, where the global expression of primary tumors, PDX models and cell lines produced three distinct groups.

Our results in PDAC and HCC confirmed previous observations made in PDAC [18] about the differences between primary tumors, cell lines and PDX models in terms of their expression profiles. This scenario highlights the influence of the cellular environment in the global expression of human primary tissue samples, cell lines and PDX models. We reasoned that the PDX-associated milieu may affect similarly to different types of human tumors, so we next investigated to what extent the gene expression of the human tumoral cells changes when engrafted in PDX models focusing on PDAC and HCC.

### Conservation of primary tumor gene expression signals in PDXs but not in cell lines

**Figure 2:**
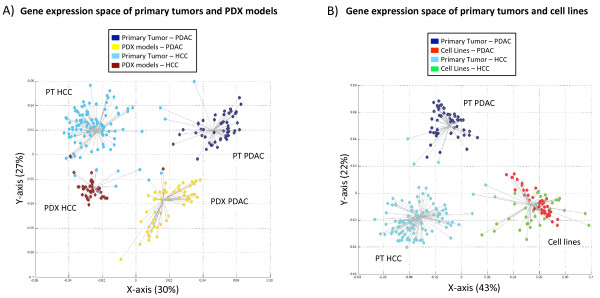
**PDAC and HCC PDX models show transcriptional differences that are not observed in tumor-derived cell lines. (A)** First two dimensions of the gene expression space of PDAC and HCC PDX models and primary tumors (see all three dimensions in Figure S3 in Additional file 1). This panel represents the result of an optimized iterative k-means clustering, where the optimal number of groups is k = 4. Each line joins a sample with the centroid of its cluster. **(B)** Gene expression space of PDAC and HCC cell lines and primary tumors. Optimal k-means clustering for k = 3. Each line joins a sample with the centroid of its cluster. The points are colored by type of sample class. See PCA results in Figure S4 and alternative clustering strategies in Figures S5 and S6 in Additional file 1.

It is reasonable to think that xenografted cells may keep a gene expression signature specific to their tumor of origin. However, if the mouse microenvironment induces similar changes in any cell type implanted into mice, then the gene expression of any cell type could acquire xenograft-specific characteristics and totally lose its original expression blueprint. We analyzed simultaneously expression profiles from HCC primary tumors, HCC PDX models, PDAC primary tumors and PDAC PDX models. Figure 2A shows the first two dimensions of the resulting tridimensional expression space (the third axis is included in Figure S3 in Additional file 1). The four-group optimal solution of the k-means clustering (CH index = 228.706) classified human primary PDAC, PDAC PDX models, human primary HCC and HCC PDX models separately. It is important to note that the clusters were obtained in an unsupervised manner (that is, sample labels were not used). Thus, we could check how many samples were well classified in their original tissue according to an automatic group selection protocol based on the expression space. We found that the groups were reliably classified; in pancreas 100% of PDAC and PDX models were grouped into their respective groups, while in liver 88% of primary HCC and 95% of HCC PDXs were correctly classified. In total, 94% of 256 total samples were correctly classified.

The first component (Figure 2A, X-axis) explained 30% of the variance and clearly separated the samples by their tissue of origin, that is, human primary tumors and PDXs from pancreas and human primary tumors and PDXs from liver clustered separately (Figure 2B). The second component (Figure 2A, Y-axis) separated PDX models and primary samples and explained 27% of the variance. The third axis explains just 8% of the variance and its incorporation in the expression space supports that there are four distinct groups of samples. Our clustering results using MCA-based expression spaces were in agreement with additional analyses using PCA (Figure S4A in Additional file 1) and hierarchical clustering (Figure S5 in Additional file 1). It is important to keep in mind that each axis of a gene expression space represents an independent source of variance, corresponding to different underlying gene expression patterns. Moreover, the sample distributions in the X-axis and Y-axis show highly significant differences (Wilcoxon’s test *P*-value = 4.1 × 10^-29^). Additionally, different clustering of the samples is obtained when using distances based on either the X-coordinates (samples grouped according to the tumor of origin; Figure S5C in Additional file 1) or the Y-coordinates (samples group according to their environment; Figure S5D in Additional file 1).

These two significant components imply the co-existence of two complementary gene expression patterns. The first component would reflect a 'gene expression memory' of the tumor of origin (similar expression calls for cells from the same origin, different calls for those from different origins). In contrast, the second component highlights the effect of an environment-associated expression signal (similar expression in similar environment, different expression otherwise). As PDXs in this second axis are grouped separately from primary tumors, this axis might represent the loss of part of the gene expression memory associated with the change of host species.

We performed a similar analysis using cell lines instead of PDX models. Figure 2B shows the resulting bi-dimensional gene expression space obtained using expression profiles from primary PDACs, PDAC cell lines, primary HCCs and HCC cell lines and which explain 65% of the variance (43% by the first axis and 22% by the second, Wilcoxon test *P*-value = 3.99 × 10^-13^). Remarkably, while PDX models derived from PDACs and HCCs form two distinct clusters (Figure 2A), the corresponding cell lines were indistinguishable (Figure 2B; Figure S6 in Additional file 1). A similar observation, regarding the higher similarity of cell lines from different origins, has been previously reported [43]. Our data suggest that, at least for PDAC and HCC, the gene expression profiles of PDXs remain partially related to the original tumors, while cell lines’ profiles are not. Our results are consistent with previous observations in breast [2], kidney [3], small cell lung cancer [44] and uveal melanomas [45] where PDXs maintain key features of the original tumors, including functional activity and gene expression profiles. In addition, Daniel *et al.*[44] found that genetic divergence between original tumors and cell lines was higher than genetic divergence between human primary tumors and PDX models.

### Contribution of non-tumoral stromal and immune infiltrating cells

Substitution of the original stroma of primary tumors by the murine stroma in PDXs is an important factor that could partially account for observed differences between primary tumors and PDXs. In fact, it is widely acknowledged that tumor samples are virtually always 'contaminated' by non-tumoral stromal and immune cells. Interestingly, PDX expression data established using human microarrays should be mostly free from the contribution of these non-tumoral components. The combination of the platform species-specificity and the well-known reduction of non-tumoral components in PDXs are expected to strongly hinder the detection of this ever-present 'contamination' in primary tumors. Consequently, the absence of this human 'non-tumoral' contamination in PDXs with respect to primary tumors could lead to an overestimation of the differences between them and primary tumors, as well as of the similarities.

**Figure 3:**
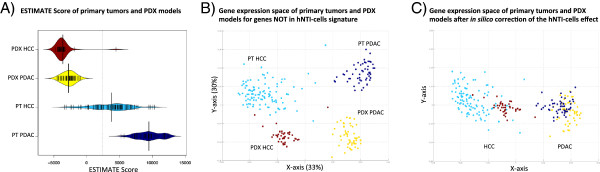
**Gene expression from tumor-infiltrating stromal and immune cells contributes to the expression space. (A)** ESTIMATE scores for each of the four groups of samples, where a higher score indicates a higher proportion of hNTI cells. **(B)** Gene expression space using the same samples as in Figure 2A, but excluding genes from the hNTI cell signature. See correlation plot between ESTIMATE score and Y-axis from Figure S7B in Additional file 1. **(C)** Gene expression space defined after *in silico* correction of the hNTI cell effect. The coordinates in each axis correspond to the residuals of the linear regression fits of each MCA component in **(B)** (see Materials and methods for details). Complementary analyses with the same samples as in Figure 2A, but using only genes from the hNTI cell signature, are shown in Figure S7 in Additional file 1. Complementary analyses using cell lines are shown in Figures S8 and S9 in Additional file 1.

To address this point, we used the ESTIMATE method [38] to infer the fraction of stromal and immune cells in the different samples. ESTIMATE is based on a gene signature characteristic of human tumor-infiltrating stromal and immune cells. According to this method, PDAC primary tumors show a higher proportion of hNTI cells than HCC primary tumors (Figure 3A). Both types of PDXs have very low (negative) ESTIMATE scores, which indicate that human non-tumoral cells are basically absent in PDXs, supporting our own experimental observations. Next, we generated a new gene expression space using the same samples as in Figure 2A but only using the 282 genes of this hNTI cell signature. Remarkably, a totally different gene expression space was obtained, where the first component is the only informative axis, explaining as much as 86% of the variance (Figure S7A in Additional file 1). Interestingly, this first MCA component separates the samples in a similar way to the ESTIMATE scores (R^2^ = 0.94, *P*-value <2.2 × 10^-16^; Figure S7B in Additional file 1).

To evaluate the effect of hNTI cells on our results, we repeated the gene expression space analysis of PDAC and HCC PDXs and primary tumors (as in Figure 2A) but excluding the probes mapping to the genes of this non-tumoral gene signature. Interestingly, removal of these 282 genes has a minor effect on the sample distribution in the resultant gene expression space (Figure 3B), showing that these genes were not the main contributors of the gene expression space. However, when we compared the Y-axis of the expression space generated without the hNTI cell signature genes (Figure 3B) with the ESTIMATE scores, we observed a striking and very significant correlation (R^2^ = 0.83, *P*-value <2.2 × 10^-16^; Figure S7D in Additional file 1; for correlation with other axes and an equivalent analysis for cell lines see Figures S7 to S9 in Additional file 1). These results indicate that differences in the contribution of hNTI cells are a main factor associated with the separated clustering of primary tumors and PDX models (Y-axis in Figure 2A and Figure 3B). These findings imply that similarities between PDX and primary tumors are strongly underestimated by gene expression experiments.

In view of this strong effect, we tried to remove (*in silico*) the contribution of non-tumoral cells to the global gene expression profiles. For this we used simple linear models for each component with ESTIMATE scores as a proxy of this contribution (see Materials and methods). After model-based removal of this trend associated with the hNTI cell signature, we generated a new gene expression space corrected for their effect (Figure 3C). In contrast to the original expression space, the corrected second component does not separate PDX models from primary tumors. This suggests that this separation was attributable to the differential contribution of hNTI cells to primary tumors and PDXs. In fact, in this corrected space, PDX models and primary PDAC samples are virtually indistinguishable, while PDXs and primary HCCs overlap and form a unique cluster.

Ideally, microdissection of the different cell types would be the best way to determine how each cell type contributes to the overall gene expression of the tissue. However, the microdissection of cells in fixed tissues is associated with higher levels of RNA degradation and the amplification of partially degraded RNA provokes potential systemic biases of gene expression [46,47]. In any case, our data also suggest that the non-tumoral (murine) infiltrating cells in PDXs have a minor contribution to the expression profiles using the Affymetrix U133 Plus 2.0 human array platform. Other studies have tried to measure the species-specificity of human microarrays in mixtures of human and mouse cells and the general conclusion was that most of the signal detected is human-specific [46,48,49]. Current approaches performing simultaneous quantification by RNAseq of mouse-specific sequence reads (stromal cells) and human-specific reads (tumoral cells) will help to better understand the contribution of the murine stroma to these changes in gene expression [48,50,51].

### Pathway analysis of PDAC PDX models

Because the gene expression of PDAC PDX models differs from that of the original PDAC tumors, it is critical to understand the similarities and differences between these two groups and, in particular, the extent to which these changes in expression can influence their validity as preclinical models of drug sensitivity [1,8,52]. Using the gene expression data, we calculated the expression frequency (number of times that each gene can be considered to be 'expressed'; see Materials and methods) of each gene in both the PDAC primary tumors and PDX models. Then, we tested if any of the biochemical and signaling pathways from the Reactome database [40] were statistically enriched in differentially expressed genes (see Pathway enrichment analyses section in Materials and methods).

**Figure 4:**
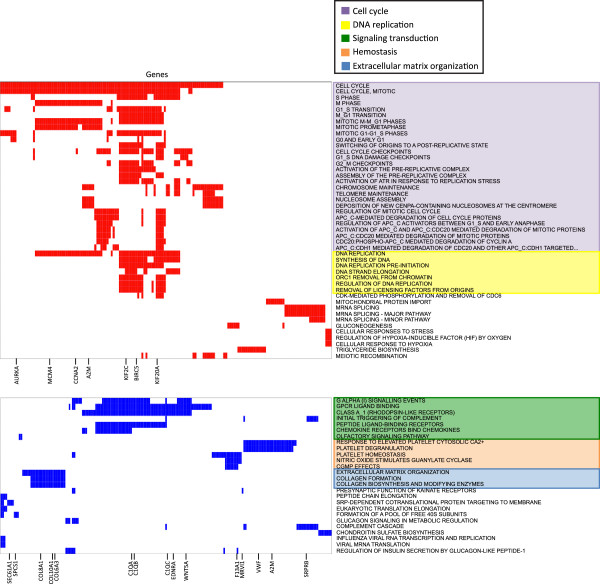
**Functional groups and pathways associated with PDX models and tumor progression.** Genes (columns) involved in 46 up-regulated pathways (rows; red), and genes (columns) involved in 26 down-regulated pathways (rows; blue). Pathways are shown on the right, with colored highlighting representing functional groups. There are two up-regulated and three down-regulated functional groups. For both up-regulated and down-regulated pathways, only the most significantly differentially expressed genes are shown. Additional file 2 lists core genes from each pathway with the expression frequency value in the PDX models and human primary tumors.

Figure 4 shows 72 pathways that presented significant accumulations of up-regulated or down-regulated genes (FDR <0.05) in PDXs. These pathways and genes represent a small number of related cellular functions, so we grouped them in functional groups. It is particularly interesting that genes in pathways related to cell cycle and DNA replication are up-regulated in PDX models while genes in pathways related to signaling transduction, hemostasis and extracellular matrix organization were significantly down-regulated (Figure 4; Additional file 2). The genes involved in cell cycle and DNA replication functional groups are mainly related to chromosome segregation and regulation of cell division subgroups. The overexpression of these genes is in agreement with the higher proliferation rates of primary tumors engrafted in mice.

Signal transduction, hemostasis and extracellular matrix organization functional groups encompass down-regulated genes in the PDX models; some genes are even common members in signal transduction and hemostasis functional groups. Interestingly, many of these functions were previously found altered in the same direction in breast PDXs [53]. Therefore, the differential expression of these genes could reflect tumor adaptation to the murine environment. However these down-regulated pathways could also reflect, to some extent, the substitution of human stroma by murine stroma. Indeed, we found that stromal genes [38] are significantly enriched in hemostasis (*P*-value = 3.1 × 10^-5^), signal transduction (*P*-value = 1.6 × 10^-7^) and extracellular matrix organization (*P*-value <10^-16^). Immune genes [38] are enriched in hemostasis (*P*-value = 0.02) and signal transduction (*P*-value = 6.5 × 10^-3^). Our results show that the functional changes detected are affected by the different contribution of hNTI cells (see above). Therefore, these and previous functional analyses of PDXs based on expression arrays should be interpreted with caution.

Many of the altered biological pathways in PDXs are typically deregulated in metastatic tumors compared with primary tumors. In fact, it has been proposed that PDX models mimic aggressive and/or metastatic tumors derived from the original primary tumors [42,46,54]. The tumor engraftment could be seen as a 'forced' metastatic situation, because the tumor needs to colonize a new environment to survive. This 'metastasis-like' phenotype of PDXs is coherent with our results, where proliferation is activated and signaling and extracellular matrix proteins change their expression profiles. To analyze this in more detail, we tested if the functional groups altered in PDX models were similarly altered in metastases (compared with primary tumors). We collected two datasets from public databases that contain data on primary pancreatic tumors and pancreatic metastases located in different organs [32,33]. Considering the metastases in groups according the colonization tissue, we performed GSEA analyses comparing each metastatic group with the corresponding primary tumors. These results are shown in Additional file 3. Although some differences were observed between colonization niches and datasets, pathways altered in PDX models were, in general, also significantly altered in metastases in the same direction. As metastatic cell clones represented only part of the primary tumor population [55], the cells that succeed engraftment in mice may actually represent one of the subpopulations of the primary tumor with higher metastatic potential. Future studies will be needed to better understand the mechanisms involved in the colonization of new niches by tumor cells, both in natural metastases in patients and in artificial PDXs. It will be particularly useful to understand if different locations of colonization may cause differences in gene expression.

### Gene expression stability through passages

**Figure 5:**
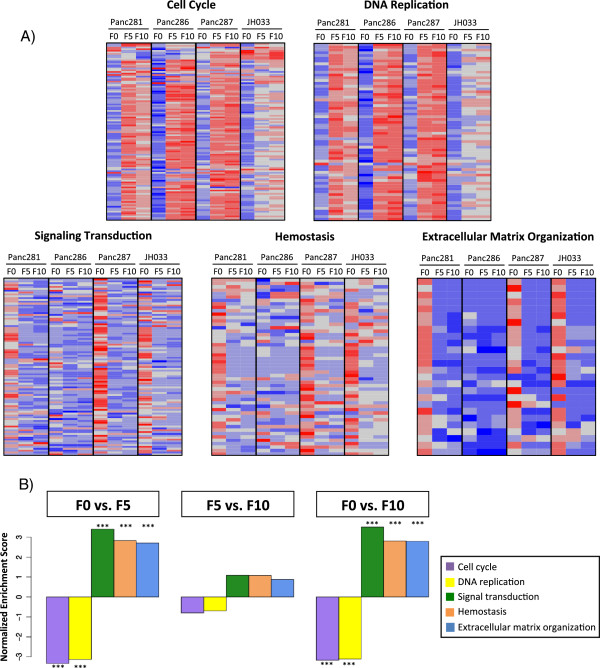
**Pathways are altered after tumor engraftment and then stable over passages. (A)** Each heatmap represents the standardized expression levels of the core genes (according to GSEA) from the functional groups significantly altered in PDX models. For each tumor we have three sampling times: F0 (passage 0, original primary tumor), F5 (fifth passage) and F10 (tenth passage). Color code: red, high; grey, medium; blue, low. **(B)** Barplot showing the GSEA normalized enrichment score (NES) for each functional group obtained in the three group comparisons: F0 versus F5 (left), F5 versus F10 (middle) and F0 versus F10 (right). The color code used is the same as in Figure 3. Significantly enriched functional groups (FDR <0.005) are indicated with three asterisks.

We analyzed gene expression profiles obtained from PDX models at different passages (Additional file 4), where passage refers to each time a tumor has been re-implanted. As each passage could accumulate more expression changes, we tested if the functional changes previously described were more pronounced after a higher number of passages. To this aim, we used a previously published dataset [42] with samples of primary tumor (F0), 5-passage PDXs (F5) and 10-passage PDXs (F10) from four patients. Using these data, we analyzed if the enriched functional groups described in the previous section (cell cycle, DNA replication, signaling transduction, hemostasis and extracellular matrix organization) showed significant differences between passages F5 and F10. Figure 5A shows the expression levels of core genes involved in the functional groups of altered pathways. Using GSEA, we confirmed that all functional groups were altered significantly after engraftment in PDX models (F0 versus F5 and F0 versus F10). However, none of the functional groups showed significant changes between F5 and F10 PDX passages (Figure 5B; Figure S10 in Additional file 1).

Taken together, these data suggest that these functional changes occur in the initial adaptation to the new environment after engraftment but no major changes occur afterwards. In other words, the gene expression pattern is stable, in agreement with previous studies in systems such as small-cell lung cancer [44], uveal melanomas [45] or colorectal cancer [52].

## Conclusions

The mouse niche environment affects tissue xenografted in mice, with some alterations in the transcriptome. The lack of immune and stromal infiltrating cells in PDXs seems to be an important contributor to these changes. Thus, caution is needed when research results using PDX models are translated to patients. Still, the transcriptomes of PDX models of PDACs and HCCs retain important aspects of their tissue of origin.

Our work has three important implications. First, PDAC (and HCC) PDX models retain gene expression similarities with the primary tumors, while cell lines do not. These results are in agreement with the better results in drug sensitivity prediction generally obtained with PDX models [11,44]. Second, some functional processes are altered in tumor cells after engraftment. Our results also show that metastases and PDX models share some functional alterations regarding primary tumors. Given that metastases frequently occur in pancreas cancer patients, our future work will focus on understanding the similarities and differences between primary and metastatic PDX models. Third, although these pathways show distinct profiles in PDX models compared with original primary tumors, they appear to be stable over passages. In our view, the stability in gene expression in PDAC PDX models over different passages is of major relevance and favors the validity of this preclinical model of disease.

## Abbreviations

FDR: false discovery rate; fRMA: frozen robust multiarray analysis; GEO: Gene Expression Omnibus; GSEA: gene set enrichment analysis; HCC: hepatocellular carcinoma; hNTI: human non-tumoral infiltrating; MCA: multiple correspondence analysis; PCA: principal component analysis; PDAC: pancreas ductal adenocarcinoma; PDX: patient-derived xenograft.

## Competing interests

The authors declare that they have no competing interests.

## Authors’ contributions

RM, DR, AV and MH were responsible for conception and design. PPL and MH coordinated the experimental work. NB and CM generated animal models. MM processed the samples for microarray hybridization. RM, AR, DJ and DR were responsible for the development of the analysis protocol. RM, DJ, DR, MH and AV were responsible for interpretation of data. RM and DR drafted the initial manuscript. All authors critically revised and approved the manuscript for publication.

## Supplementary Material

Additional file 1 Supplementary figures. Click here for file

Additional file 2: Table S1 Core genes from each pathway with the expression frequency value in the PDX models and human primary tumors and presence or absence in each functional group. Click here for file

Additional file 3: Table S2 GSEA results for pathways altered in PDX models in metastases. Table contains FDR values of functional groups for each comparison between primary tumor and different metastases obtained from two datasets [32,33]. Click here for file

Additional file 4: Table S3 Information about origin of PDXs and microarray hybridization, the publication status of expression data and number of passages of PDX models. Click here for file
