# Supplementary material for: Transcriptional dissection of pancreatic tumors engrafted in mice
Source: Genome Med. 2014 Apr 16;6(4):27. doi: 10.1186/gm544 (PMC4062047; doi:10.1186/gm544)
Supplement: Additional file 1 — Supplementary figures. [file gm544-S1.pdf]

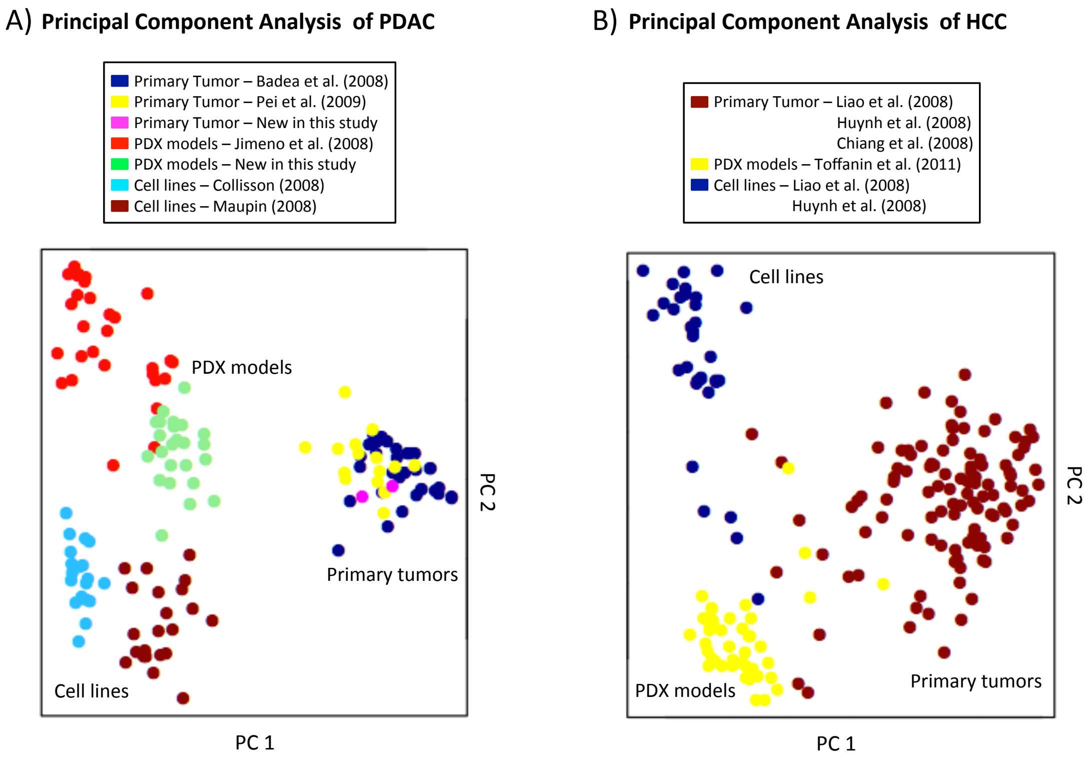

Figure S1. Principal Component Analysis results. **(A)** PCA with expression intensities from PDAC samples (samples from Figure 1A). **(B)** PCA with expression intensities from HCC samples (samples from Figure 1B).

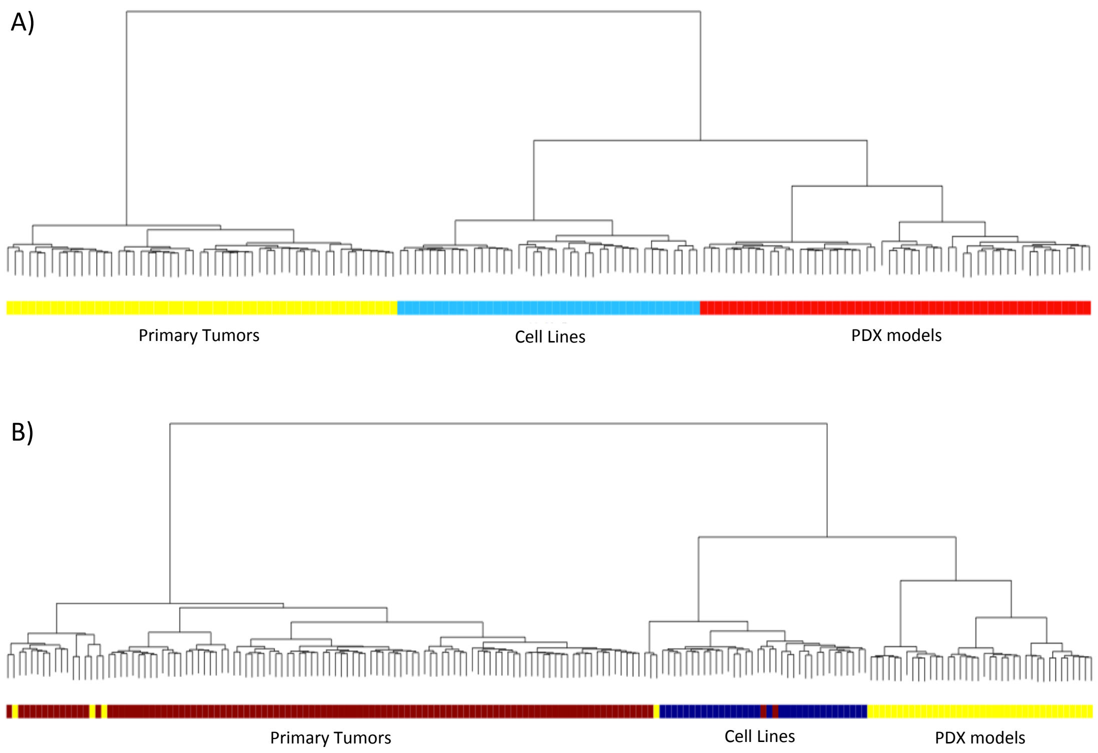

Figure S2. Hierarchical clustering. **(A)** Hierarchical clustering using PDAC samples from Figure 1A. **(B)** Hierarchical clustering using HCC samples from Figure 1B.

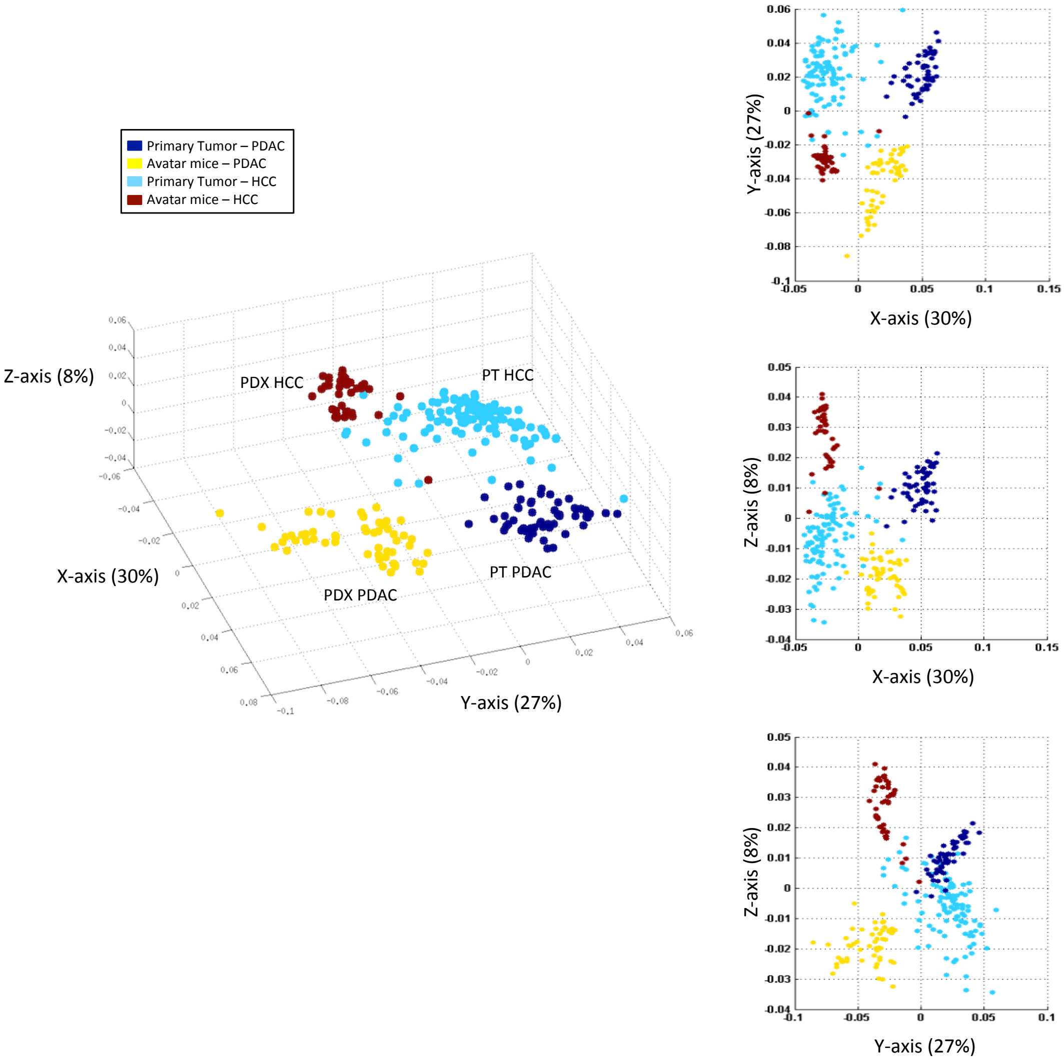

Figure S3. Tridimensional view of gene expression space of Figure 2A. Our protocol establishes three axes as informative in this gene expression space. On the left we show a 3D view of the expression space and on the right we show the different combinations of three coordinates. The third axis, Z-axis, explains 8% of the variance (Wilcoxon's test p-value = 6.5×10^{-4}).

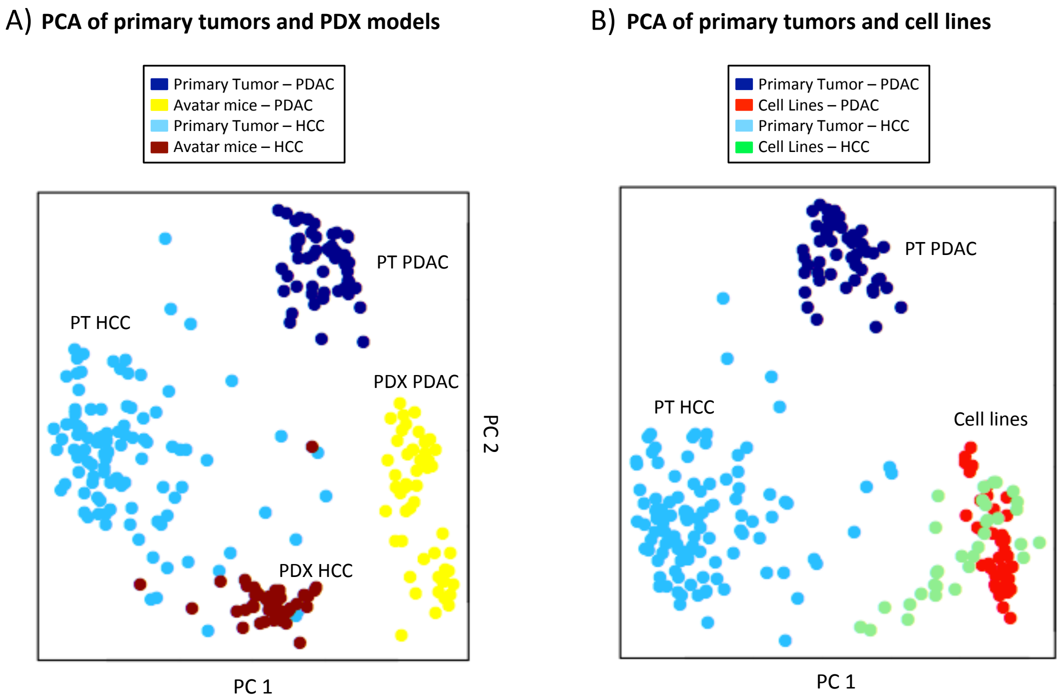

Figure S4. Principal Component Analysis results. **(A)** PCA with expression intensities from primary tumors and PDX models (samples from Figure 2A). **(B)** PCA with expression intensities from primary tumors and cell lines (samples from Figure 2B).

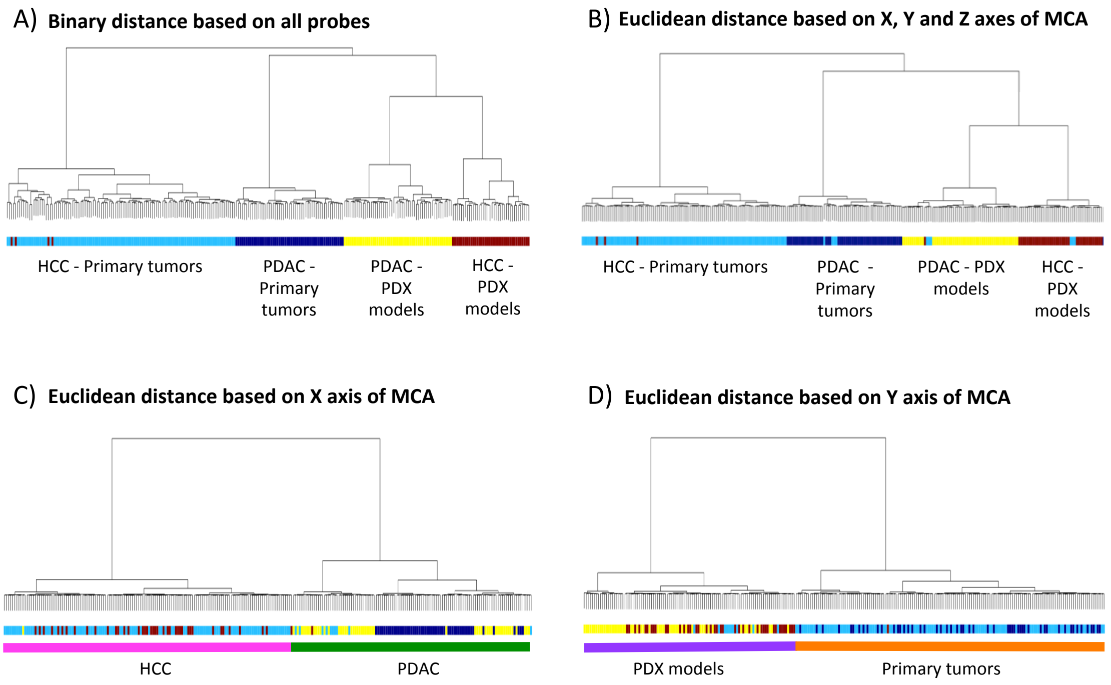

Figure S5. Hierarchical clustering of primary tumor and PDX models. **(A)** Hierarchical clustering of primary tumor and PDX models based on binary distance of expression calls of all probes. **(B)** Hierarchical clustering of primary tumor and PDX models based on Euclidean distance from X, Y and Z axes obtained in MCA of Figure 2A. **(C)** Hierarchical clustering of primary tumor and PDX models based on Euclidean distance from X-axis obtained in MCA of Figure 2A. **(D)** Hierarchical clustering of primary tumor and PDX models based on Euclidean distance from Y-axis obtained in MCA of Figure 2A. **(A-D)** Color code: Dark blue correspond to PDAC, yellow are PDAC PDX models, light blue are HCC and dark red are HCC PDX models. In figure C and D there are a second color bar to facilitate the visualization of the clusters.

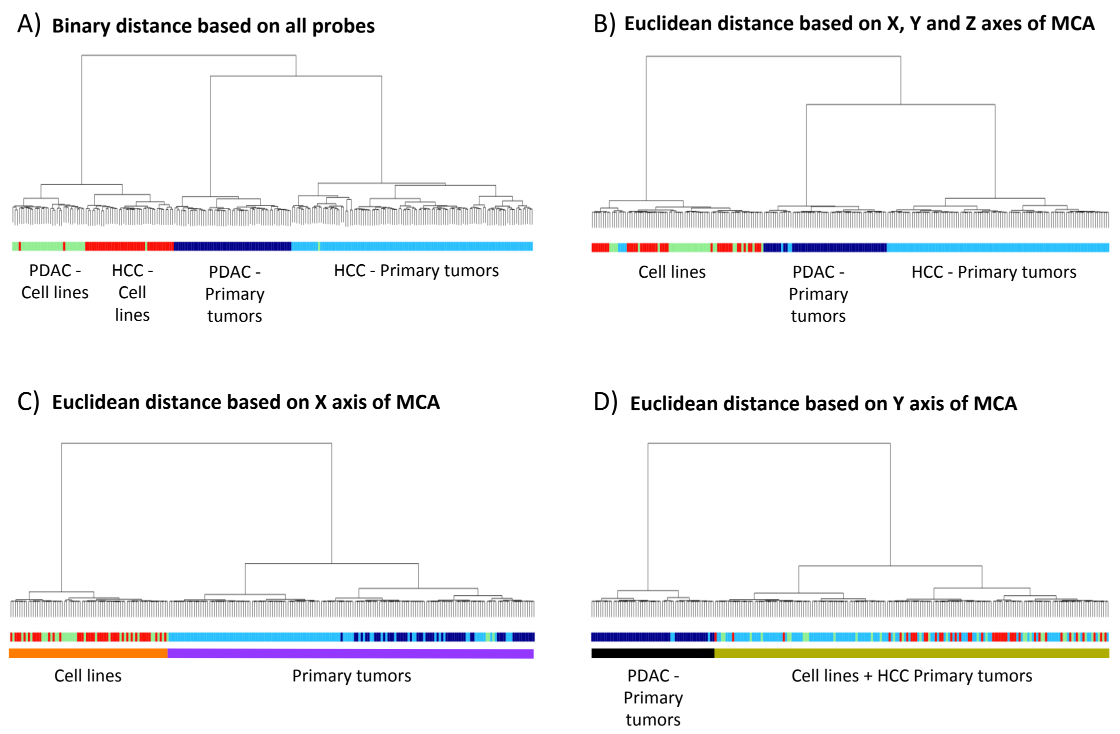

Figure S6. Hierarchical clustering of primary tumor and cell lines. **(A)** Hierarchical clustering of primary tumor and cell lines based on binary distance of expression calls of all probes. **(B)** Hierarchical clustering of primary tumor and cell lines based on Euclidean distance from X and Y axes obtained in MCA of Figure 2B. **(C)** Hierarchical clustering of primary tumor and cell lines based on Euclidean distance from X-axis obtained in MCA of Figure 2B. **(D)** Hierarchical clustering of primary tumor and cell lines based on Euclidean distance from Y-axis obtained in MCA of Figure 2B. **(A-D)** Color code: Dark blue correspond to PDAC, red are PDAC cell lines, light blue are HCC and green are HCC cell lines. In figure C and D there are a second color bar to facilitate the visualization of the clusters.

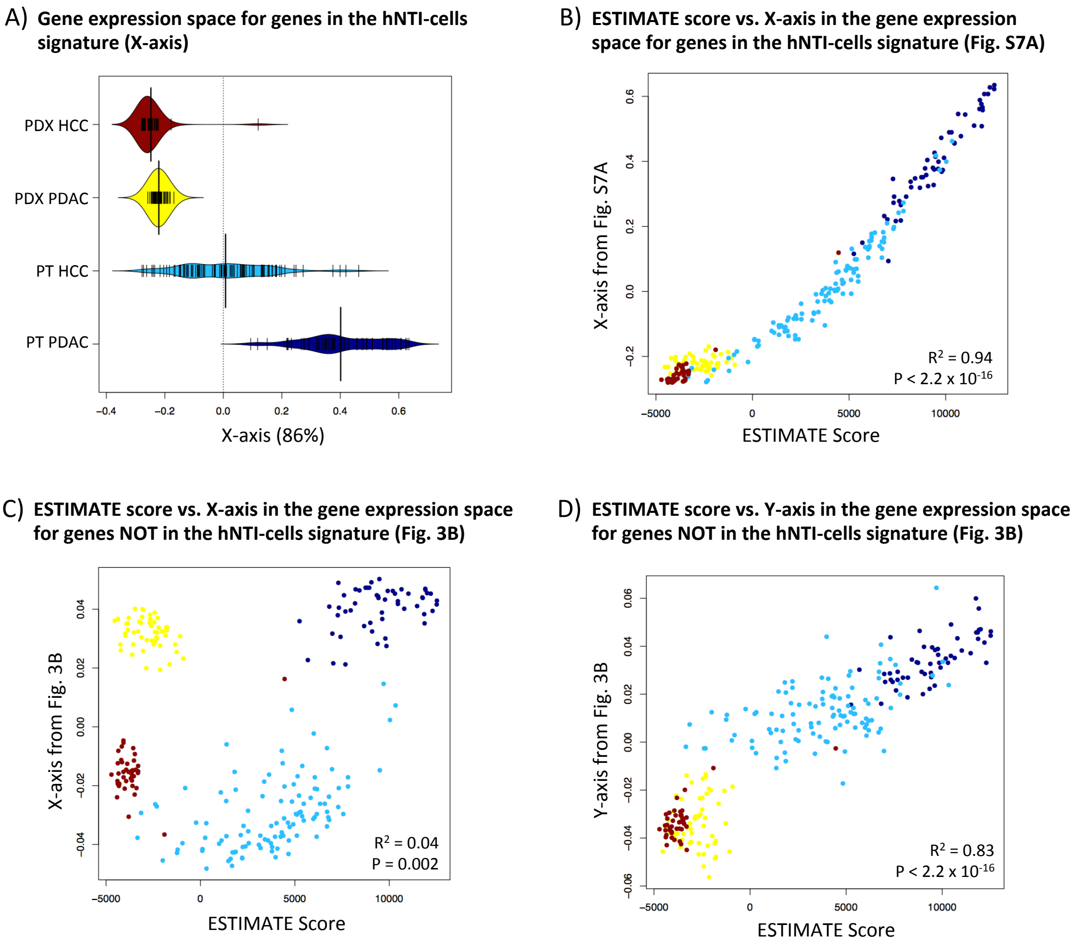

Figure S7. Contribution of hNTI-cells in primary tumors and PDX samples. **(A)** X coordinates from MCA obtained when only genes in the hNTI-cells signature are used (we represented X coordinates because the second axis of the MCA was not statistically informative). **(B)** Correlation plot between ESTIMATE Score and X-axis from Figure S7A (X coordinates from MCA using only probes mapping the genes of hNTI-cells signature). **(C)** Correlation plot between ESTIMATE Score and X-axis from Figure 3B (gene expression space performed excluding the probes mapping to the genes of hNTI-cells signature). **(D)** Correlation plot between ESTIMATE Score and Y-axis from Figure 3B (gene expression space performed excluding the probes mapping to the genes of hNTI-cells signature). **(A-D)** Color code: Dark blue correspond to PDAC, yellow are PDAC PDX models, light blue are HCC and dark red are HCC PDX models.

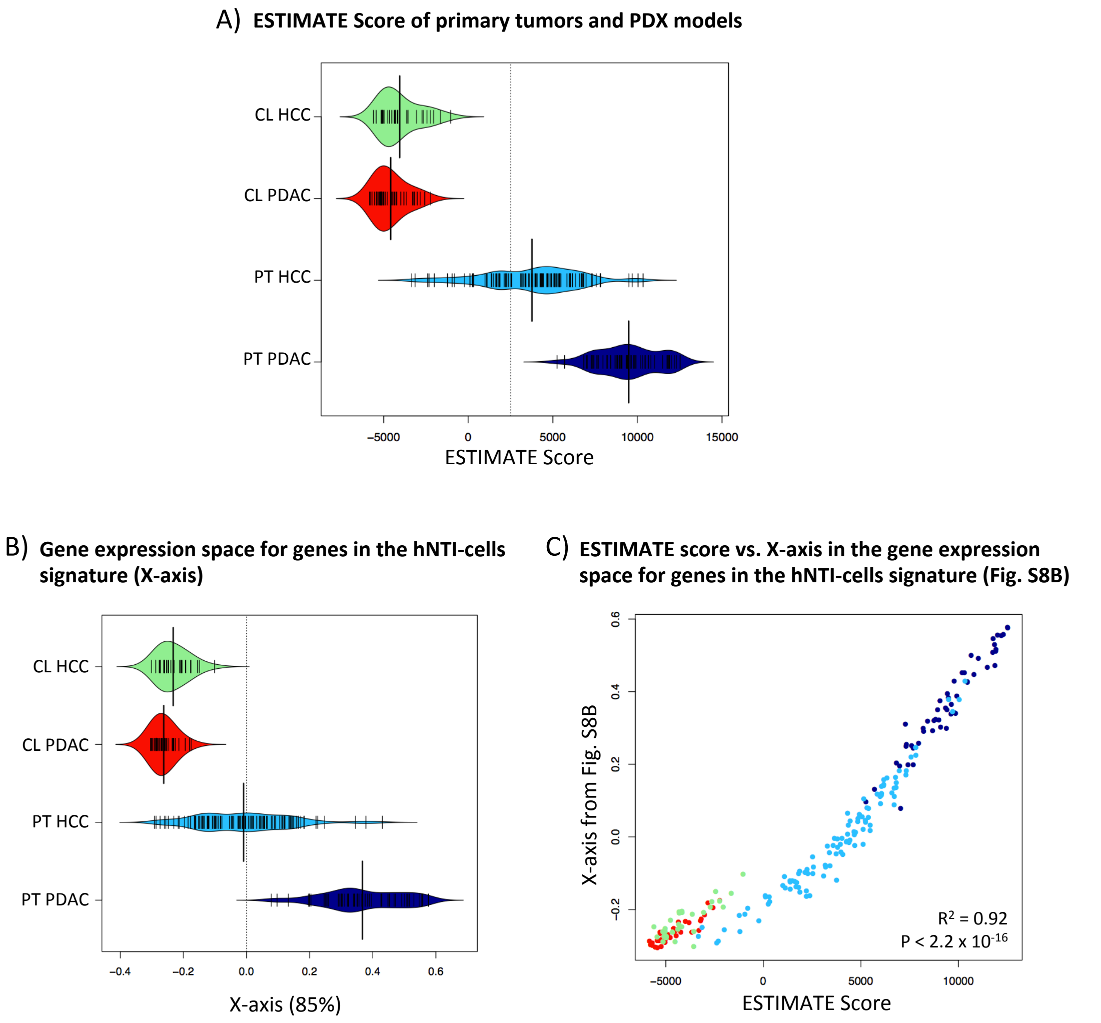

Figure S8. Contribution of hNTI-cells in primary tumors and cell lines. **(A)** ESTIMATE scores for each of the four groups of samples, where higher score indicates a higher proportion of infiltrating cells. **(B)** X coordinates from MCA obtained when only genes in the hNTI-cells signature are used (we represented X coordinates because the second axis of the MCA was not statistically informative). **(C)** Correlation plot between ESTIMATE Score and X-axis from Figure S8B (X coordinates from MCA using only probes mapping the genes of hNTI-cells signature). **(A-C)** Color code: Dark blue correspond to PDAC, red are PDAC cell lines, light blue are HCC and green are HCC cell lines.

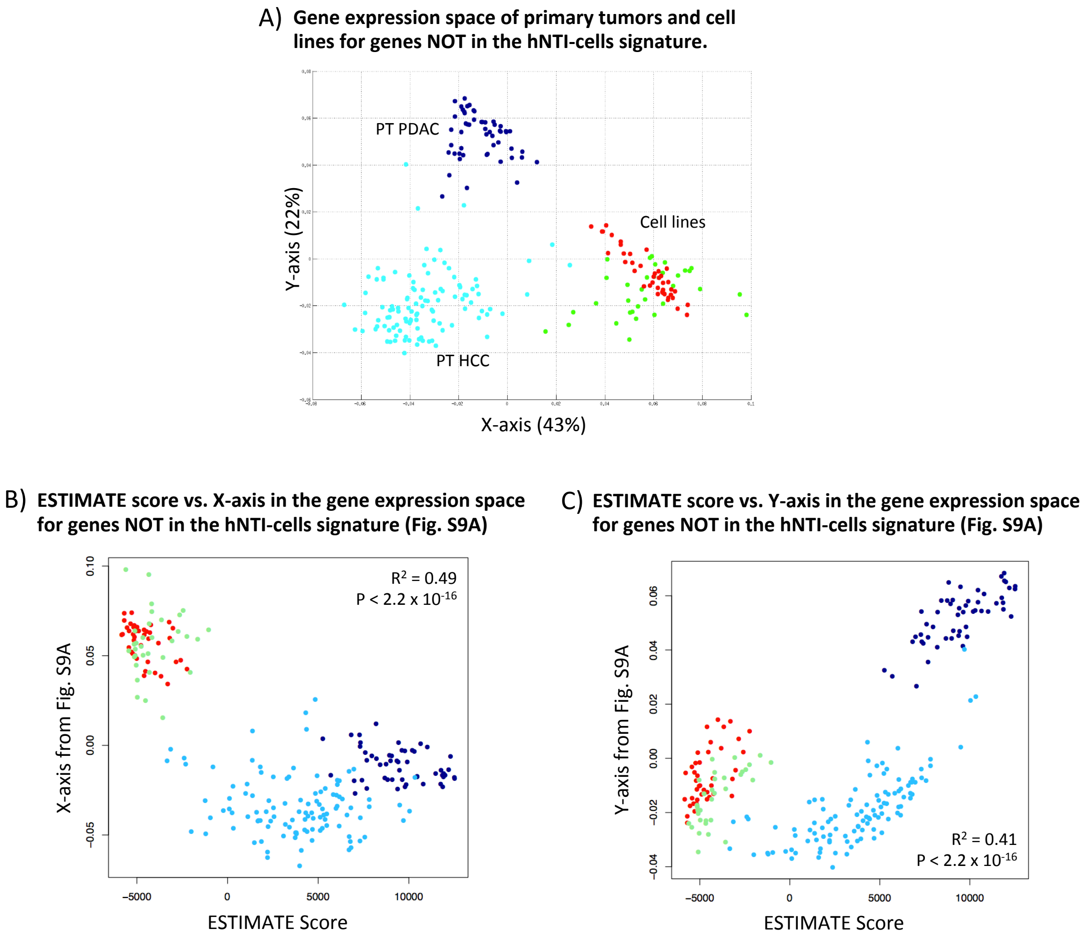

Figure S9. Contribution of all probes excluding those mapping of hNTI-cells signature in primary tumors and cell lines. **(A)** Gene expression space using the same samples of Figure 2B, but excluding genes from the hNTI-cells signature. **(B)** Correlation plot between ESTIMATE Score and X-axis from Figure S9A (gene expression space performed excluding the probes mapping to the genes of hNTI-cells signature). **(C)** Correlation plot between ESTIMATE Score and Y-axis from Figure S9A (gene expression space performed excluding the probes mapping to the genes of hNTI-cells signature). **(A-D)** Color code: Dark blue correspond to PDAC, red are PDAC cell lines, light blue are HCC and green are HCC cell lines.

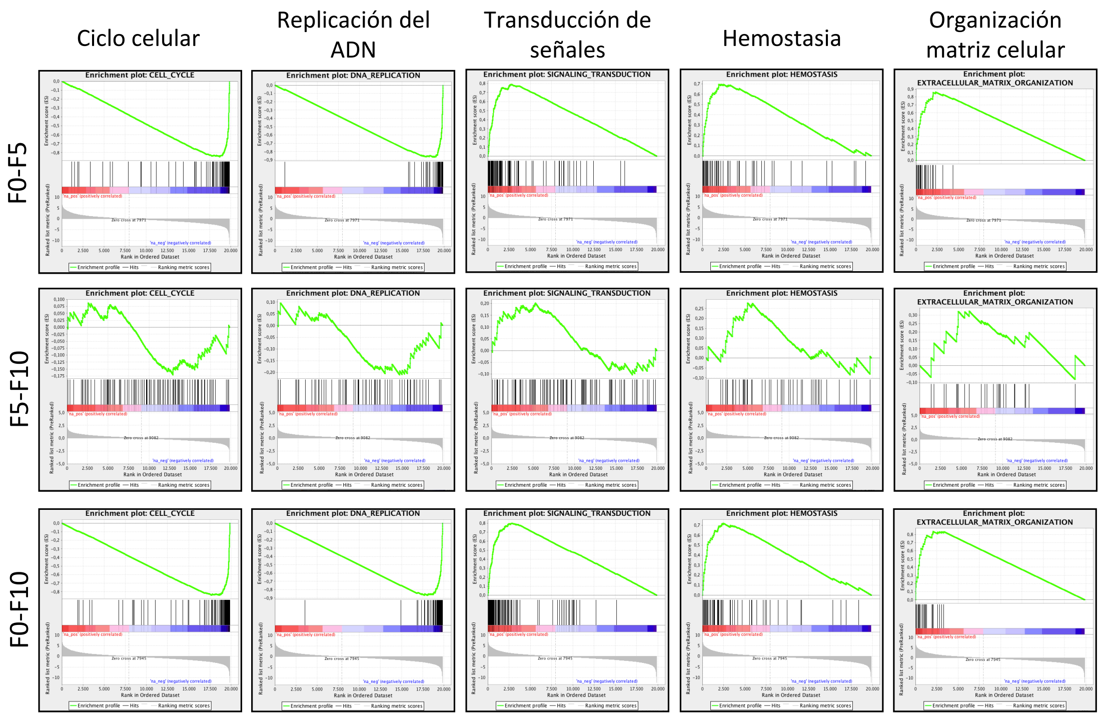

Figure S10. Enrichment plots of functional groups. Rows are different comparisons between generations. Columns are different functional groups. Comparisons F0-F5 and F0-F10 are significant (FDR < 0.05). F5-F10 is not significant in all functional groups. This is complementary to Figure 5B.
